# A Polymorphism of *ORAI1* rs7135617, Is Associated with Susceptibility to Rheumatoid Arthritis

**DOI:** 10.1155/2014/834831

**Published:** 2014-04-08

**Authors:** Jeng-Hsien Yen, Che-Mai Chang, Yu-Wen Hsu, Chih-Hung Lee, Mei-Shin Wu, Daw-Yang Hwang, Ben-Kuen Chen, Hsien-Tzung Liao, Man-Tzu Marcie Wu, Wei-Chiao Chang

**Affiliations:** ^1^College of Medicine, Kaohsiung Medical University, and Department of Internal Medicine, Kaohsiung Medical University Hospital, Kaohsiung 83301, Taiwan; ^2^Department of Clinical Pharmacy, School of Pharmacy, Taipei Medical University, Taipei 11031, Taiwan; ^3^Department of Dermatology, Kaohsiung Chang Gung Memorial Hospital, Kaohsiung 83301, Taiwan; ^4^Chang Gung University College of Medicine, Taoyuan 33302, Taiwan; ^5^Division of Nephrology, Kaohsiung Medical University Hospital, Kaohsiung Medical University, Kaohsiung 80756, Taiwan; ^6^Institute of Bioinformatics and Biosignal Transduction, College of Bioscience and Biotechnology, National Cheng Kung University, Tainan 701, Taiwan; ^7^Division of Allergy, Immunology and Rheumatology, Department of Internal Medicine, Taipei Medical University-Wan Fang Hospital, Taipei 11696, Taiwan; ^8^Department of Internal Medicine, School of Medicine, College of Medicine, Taipei Medical University, Taipei 11696, Taiwan; ^9^Department of Pharmacy, Taipei Medical University-Wan Fang Hospital, Taipei 11696, Taiwan; ^10^Master Program for Clinical Pharmacogenomics and Pharmacoproteomics, School of Pharmacy, Taipei Medical University, Taipei 11031, Taiwan; ^11^Graduate Institute of Clinical Medicine, College of Medicine, Kaohsiung Medical University, Kaohsiung 80708, Taiwan

## Abstract

Rheumatoid arthritis (RA), a chronic inflammatory disease usually occurring in synovial tissues and joints, is highly associated with genetic and environmental factors. *ORAI1*, a gene related to cellular immune system, has been shown to be involved in the pathogenesis of chronic inflammatory diseases and immune diseases. To identify whether *ORAI1* gene contributes to RA susceptibility, we enrolled 400 patients with RA and 621 healthy individuals for a case-control genetic association study. Five tagging single nucleotides polymorphisms (tSPNs) within *ORAI1* gene were selected for genotyping. An SNP, rs7135617, showed a significant correlation with the risk of RA. Our results indicated that genetic polymorphism of *ORAI1* gene is involved in the susceptibility of RA in a Taiwanese population.

## 1. Introduction

Rheumatoid arthritis (RA) is an autoimmune disease that affects joints in the body. RA is also a chronic inflammatory disease that can lead to long-term joint damage, chronic pain, and loss of motor function in the hands. RA frequently affects smaller joints [1]. Symptoms caused by RA include joint stiffness, a low-grade fever, rheumatoid nodules, and lumps of tissue under the skin. The prevalence of RA is 0.5%~1%, which is relatively constant in many populations [1]. A high prevalence of RA was reported in Indians; in contrast, a low prevalence of RA was observed in Chinese and Japanese populations [1]. Differences of RA prevalence among populations reveal the importance of genetic factors in the risk of RA.

The cause of RA is still unclear. The immune system plays an important role in RA. Several genetic regions were reported to be associated with RA. The major histocompatibility complex (MHC) is a well-known region [2]. HLA DRB1 alleles were shown to be significant markers of RA in several populations [3–8]. In addition, using a genome-wide association study, Kochi et al. identified a polymorphism in a gene encoding chemokine (C-C motif) receptor 6 (CCR6) at 6q27, which was associated with RA [9]. The contribution of this region is estimated to be about 30% of the total genetic effects on RA susceptibility. This regulatory variant in* CCR6* was further confirmed in Taiwanese RA patients [10].

The store-operated calcium channel plays an important role in activation of T-lymphocytes. Orai1 is the pore-forming subunit of the store-operated calcium channel [11]. A loss of functional mutation of *ORAI1* was found to cause severe combined immunodeficiency (SCID) [12]. Genetic polymorphisms of ORAI1 were reported to be associated with a risk of HLA-B27-positive ankylosing spondylitis [13]. However, the role of ORAI1 in RA is still unclear. In this study, we assessed whether genetic variations in* ORAI1* contribute to RA susceptibility in the Taiwanese population.

## 2. Materials and Methods

### 2.1. Study Subjects

In total, 1021 Taiwanese individuals including 400 patients with rheumatoid arthritis (RA) and 621 healthy subjects were enrolled at Kaohsiung Medical University Hospital. Patients with RA were diagnosed to fulfill the revised criteria of the American Rheumatism Association for RA. This study was approved by the Institutional Review Board of Kaohsiung Medical University Hospital. All participants were provided with sufficient information and a consent form for the study before clinical data and samples were collected.

### 2.2. DNA Extraction and Genotyping

Patients' genomic DNAs were isolated from whole blood samples using a Gentra extraction kit and ethanol precipitation as described in our previous study [14]. Genotyping for single-nucleotide polymorphisms (SNPs) of* Orai1* was conducted using a TaqMan Allelic Discrimination Assay (Applied Biosystems, Foster City, CA). A polymerase chain reaction (PCR) was performed in a 96-well microplate with an ABI 9700 thermal cycler (Applied Biosystems, Foster City, CA). After the PCR, the fluorescence was measured and analyzed using system SDS software version 1.2.3 (Applied Biosystems, Foster City, CA).

### 2.3. Statistical Analysis

JMP 8.0 software for Windows (SAS Institute, Cary, NC) was used for the statistical analysis of genotyping results. Statistical differences in genotypes and allelic frequencies between cases and controls were assessed using a *χ* ^2^ test. A linkage disequilibrium (LD) map used to define the haplotype blocks was constructed using Haploview software (version 4.2; http://www.broad.mit.edu/mpg/haploview/). The haplotype analysis was performed to compare distributions of haplotype frequencies of* ORAI1* between cases (RA) and controls.

## 3. Results

### 3.1. Clinical Characteristics of Subjects

To investigate whether SNPs of* ORAI1* contribute to the susceptibility to RA, we performed a case and control association study. As shown in the Table 1, 400 rheumatoid arthritis patients and 621 healthy controls were recruited. Of the RA patients, 82.2% were female. The mean age was 62.4 years. In the healthy controls, 57.5% individuals were female and the overall mean age was 51.2 years.

### 3.2. A Significant Association between rs7135617 and Susceptibility of RA

In this study, five tagged SNPs (tSNPs) of* ORAI1* (rs12320939, rs12313273, rs7135617, rs6486795, and rs712853) with minor allele frequencies (MCFs) of >10% were selected from the HapMap Han Chinese database. Differences in genotypic and allelic frequencies of SNPs between cases and controls were compared. As shown in Table 2, rs7135617 revealed a significant association with RA in both the genotypic (*P* = 0.004) and recessive models (odds ratio (95% CI): 1.58 (1.14~2.19); *P* = 0.006).

### 3.3. Haplotype Analysis of *ORAI1* Genetic Polymorphisms in the Susceptibility to RA

**Figure 1:**
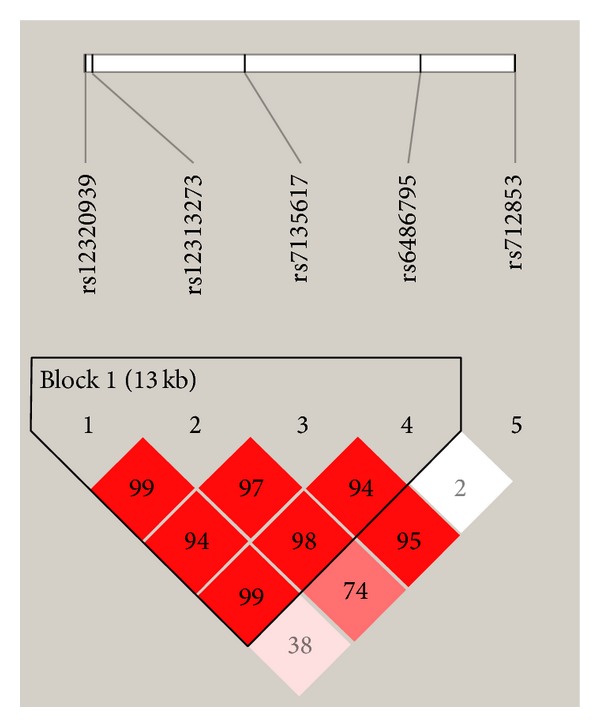
Five tSNPs on the LD map of* ORAI1* gene.

To further identify whether haplotypes of* ORAI1* were correlated with RA, we created an LD map (Figure 1) and analyzed haplotype frequency differences between RA patients and controls. The haplotype analysis showed that no association was observed in pairwise allelic comparisons of rs12313273/rs7135617 (Table 3) or rs7135617/rs6486795 (Table 4).

## 4. Discussion

In this study, we screened SNPs of* ORAI1* and performed a case-control association study. In this study, 1021 subjects (400 cases and 621 controls) were recruited. Five genetic polymorphisms were selected for genotyping. Our results indicated a significant association between rs7135617 and susceptibility to RA. Previous studies reported significant associations between genetic polymorphisms of* ORAI1* and inflammatory diseases such as ankylosing spondylitis, calcium nephrolithiasis, and atopic dermatitis [13, 15, 16]. In this study, we found an SNP (rs7135617) located in the intron of* ORAI1* associated with a risk of RA in the Taiwanese population.

*ORAI1*-mediated calcium signaling was reported to be involved in a variety of human diseases. Feske et al. identified a mutation in* ORAI1* from SCID patients [12]. A missense mutation resulted in the dysfunction of store-operated calcium entry that in turn attenuated immune responses [12]. Our previous studies indicated that ORAI1 was highly expressed in the spleen, an organ involved in immune system [16]. The rs7135617 within* ORAI1* was associated with an autoimmune disease, ankylosing spondylitis. Consistent with a previous report, this study also confirmed an important role of ORAI1 polymorphism rs7135617 in RA. However, functional role of “intronic splicing regulatory elements of* ORAI1*” underlying RA susceptibility is not clear. Therefore, we further applied Human Splicing Finder version 2.4.1 (HSF) [17] to analyze the possible functions of rs7135617G>T. Results indicated that rs7135617 was predicted as a potential target binding site of SR SC35 protein. SR SC35 protein is an important splicing factor which can influence selection of splice site [18]. The consensus value of rs7135617 wild-type (G) motif is 75.97 whereas the mutant-type (T) motif is 91.09. The variation of the consensus value (ΔCV) is +19.9%. A higher consensus value indicates higher strength and more possibility to be the splicing enhancer binding motif of SC35 protein. Combined with bioinformatics findings and genotyping data, our results imply that* ORAI1* polymorphism rs7135617 may influence splicing process which further affects calcium signaling.

This study has some limitations. First, the collection of samples did not contain clinical biochemical data of RA patients. Therefore, this study was only able to detect associations between SNPs and the risk of RA. Second, rs7135617 is located in the intron. The T allele is a risk allele for RA. However, further functional role of* ORAI1 *polymorphism rs7135617 requires experimental validation in order to clarify the mechanism underlying calcium signaling and susceptibility of RA.

Third, the study was limited by the modest sample size (1021 subjects); however, we believe this might be partly overcome by the fact that our samples are homogeneous and well defined in terms of phenotype assessment.

Given the polygenic nature of immune diseases such as RA, the susceptibility gene* ORAI1* could provide new clues to the pathogenesis of RA. Although a larger-scale population study is needed, our results, at least in part, indicated an important role of* ORAI1* gene in the susceptibility to RA. Further study of the relationship between* ORAI1* genotypes and the downstream functional relevance during chronic inflammation of the joints should be conducted in order to understand the etiology of RA.

## Figures and Tables

**Table 1:** Basal characteristics of patients with rheumatoid arthritis (RA) and of normal controls.

Characteristics	Patients with RA	Normal control
Number of subjects	400	621
Gender: female, no. (%)	329 (82.2%)	357 (57.5%)
Age (years)	62.4 ± 13.4	51.2 ± 16.2
Range (years)	22–90	11–88

**Table 2:** Genotyping and allele frequency of *ORAI1* gene in rheumatoid arthritis patients and normal controls.

	Genotype	Case (%) (*n* = 400)	Control subjects (%) (*n* = 621)	Allele	Case (%) (*n* = 400)	Control subjects (%) (*n* = 621)	Genotype *P* value	Recessive *P* value	Allelic *P* value
rs12320939	TT	95 (24.4)	144 (23.4)	T	382 (49.1)	602 (48.9)	0.868	0.715	0.945
	TG	192 (49.4)	314 (51.1)	G	396 (50.9)	628 (51.1)		1.06	1.01
	GG	102 (26.2)	157 (25.5)					(0.79–1.42)	(0.84–1.20)

rs12313273	CC	28 (7.8)	54 (8.8)	C	202 (28.0)	355 (28.9)	0.850	0.573	0.660
	CT	146 (40.4)	247 (40.2)	T	520 (72.0)	873 (71.1)		0.87	0.96
	TT	187 (51.8)	313 (51.0)					(0.54–1.40)	(0.78–1.17)

rs7135617	TT	83 (22.5)	96 (15.5)	T	318 (43.1)	505 (40.9)	0.004*	0.006*	0.331
	TG	152 (41.2)	313 (50.7)	G	420 (40.9)	731 (59.1)		1.58	1.10
	GG	134 (36.3)	209 (33.8)					(1.14–2.19)	(0.91–1.32)

rs6486795	CC	57 (14.9)	82 (13.3)	C	291 (38.1)	464 (37.7)	0.687	0.475	0.849
	CT	177 (46.3)	300 (48.7)	T	473 (61.9)	768 (62.3)		1.14	1.02
	TT	148 (38.7)	234 (38.0)					(0.79–1.65)	(0.85–1.23)

rs712853	CC	37 (9.7)	64 (10.6)	C	238 (31.1)	396 (32.7)	0.740	0.643	0.442
	CT	164 (42.8)	268 (44.3)	T	528 (68.9)	814 (67.3)		0.90	0.93
	TT	182 (47.5)	273 (45.1)					(1.38–0.59)	(1.13–0.76)

*Significant (*P* < 0.05) values are in bold.

**Table 3:** Haplotype frequencies of the *ORAI1* gene in rheumatoid arthritis patients and normal controls patients.

rs12313273/rs7135617	Case (%) (*n* = 400)	Control subjects (%) (*n* = 621)	OR (95% CI)	*P* value
T/T	304 (42.0)	501 (40.8)	1.09 (0.87–1.37)	0.4512
T/G	220 (30.4)	372 (30.3)	1.06 (0.84–1.35)	0.6210
C/G	197 (27.2)	354 (28.8)	Reference	

Haplotype frequency less than 1% was excluded.

**Table 4:** Haplotype frequencies of the *ORAI1* gene in rheumatoid arthritis patients and normal controls.

rs7135617/rs6486795	Case (%) (*n* = 400)	Control subjects (%) (*n* = 621)	OR (95% CI)	*P* value
T/T	295 (41.1)	501 (40.7)	1.00 (0.81–1.23)	0.9961
G/T	142 (19.8)	265 (21.5)	0.91 (0.71–1.17)	0.4629
G/C	271 (37.7)	460 (37.4)	Reference	

Haplotype frequency less than 1% was excluded.

## References

[1] Silman AJ, Pearson JE (2002). Epidemiology and genetics of rheumatoid arthritis. *Arthritis Research*.

[2] Deighton CM, Walker DJ, Griffiths ID, Roberts DF (1989). The contribution of HLA to rheumatoid arthritis. *Clinical Genetics*.

[3] Wakitani S, Murata N, Toda Y (1997). The relationship between HLA-DRB1 alleles and disease subsets of rheumatoid arthritis in Japanese. *British Journal of Rheumatology*.

[4] del Rincon I, Escalante A (1999). HLA-DRB1 alleles associated with susceptibility or resistance to rheumatoid arthritis, articular deformities, and disability in Mexican Americans. *Arthritis and Rheumatism*.

[5] Balsa A, Minaur NJ, Pascual-Salcedo D (2000). Class II MHC antigens in early rheumatoid arthritis in Bath (UK) and Madrid (Spain). *Rheumatology*.

[6] Zanelli E, Breedveld FC, de Vries RRP (2000). HLA class II association with rheumatoid arthritis: facts and interpretations. *Human Immunology*.

[7] Pascual M, Nieto A, Lopez-Nevot MA (2001). Rheumatoid arthritis in southern Spain: toward elucidation of a unifying role of the HLA class II region in disease predisposition. *Arthritis and Rheumatism*.

[8] Citera G, Padulo LA, Fernandez G, Lazaro MA, Rosemffet MG, Maldonado Cocco JA (2001). Influence of HLA-DR alleles on rheumatoid arthritis: susceptibility and severity in Argentine patients. *Journal of Rheumatology*.

[9] Kochi Y, Okada Y, Suzuki A (2010). A regulatory variant in CCR6 is associated with rheumatoid arthritis susceptibility. *Nature Genetics*.

[10] Chang WC, Woon PY, Wei JC (2012). A single-nucleotide polymorphism of CCR6 (rs3093024) is associated with susceptibility to rheumatoid arthritis but not ankylosing spondylitis, in a Taiwanese population. *The Journal of Rheumatology*.

[11] Zhou Y, Ramachandran S, Oh-hora M, Rao A, Hogan PG (2010). Pore architecture of the ORAI1 store-operated calcium channel. *Proceedings of the National Academy of Sciences of the United States of America*.

[12] Feske S, Gwack Y, Prakriya M (2006). A mutation in Orai1 causes immune deficiency by abrogating CRAC channel function. *Nature*.

[13] Wei JC-C, Yen J-H, Juo S-HH (2011). Association of ORAI1 haplotypes with the risk of HLA-B27 positive ankylosing spondylitis. *PLoS ONE*.

[14] Yang KD, Chang J-C, Chuang H (2010). Gene-gene and gene-environment interactions on IgE production in prenatal stage. *Allergy*.

[15] Chou Y-H, Juo S-HH, Chiu Y-C (2011). A polymorphism of the ORAI1 gene is associated with the risk and recurrence of calcium nephrolithiasis. *The Journal of Urology*.

[16] Chang W-C, Lee C-H, Hirota T (2012). ORAI1 genetic polymorphisms associated with the susceptibility of atopic dermatitis in Japanese and Taiwanese populations. *PLoS ONE*.

[17] Desmet F-O, Hamroun D, Lalande M, Collod-Bëroud G, Claustres M, Béroud C (2009). Human splicing finder: an online bioinformatics tool to predict splicing signals. *Nucleic Acids Research*.

[18] Liu H-X, Chew SL, Cartegni L, Zhang MQ, Krainer AR (2000). Exonic splicing enhancer motif recognized by human SC35 under splicing conditions. *Molecular and Cellular Biology*.

